# A Novel Heterozygous Mutation c.1627G>T (p.Gly543Cys) in the *SLC34A1* Gene in a Male Patient with Recurrent Nephrolithiasis and Early Onset Osteopenia: A Case Report

**DOI:** 10.3390/ijms242417289

**Published:** 2023-12-09

**Authors:** Francesca Giusti, Francesca Marini, Hatim Al-alwani, Elena Marasco, Paolo Garagnani, Aliya A. Khan, Maria Luisa Brandi

**Affiliations:** 1 Donatello Bone Clinic, Villa Donatello Hospital, 50019 Sesto Fiorentino, Italy; 2 Department of Experimental and Clinical Biomedical Sciences, University of Florence, 50139 Florence, Italy; 3 Fondazione FIRMO Onlus, Italian Foundation for the Research on Bone Diseases, 50129 Florence, Italy; 4 Divisions of Endocrinology and Metabolism and Geriatrics, McMaster University, Hamilton, ON L8S 4L8, Canadadraliyakhan@gmail.com (A.A.K.); 5 Personal Genomics SRL, 37136 Verona, Italy; 6 Department of Medical and Surgical Sciences (DIMEC), Alma Mater Studiorum University of Bologna, 40126 Bologna, Italy; 7 IRCCS Azienda Ospedaliero-Universitaria di Bologna, 40138 Bologna, Italy; 8 IRCCS San Raffaele Hospital, 20132 Milano, Italy

**Keywords:** *SLC34A1* gene, autosomal dominant hypophosphatemic nephrolithiasis/osteoporosis type 1, genetic testing, gene mutation, recurrent nephrolithiasis, bone mass loss

## Abstract

Serum phosphate concentration is regulated by renal phosphate reabsorption and mediated by sodium–phosphate cotransporters. Germline mutations in genes encoding these cotransporters have been associated with clinical phenotypes, variably characterized by hyperphosphaturia, hypophosphatemia, recurrent kidney stones, skeletal demineralization, and early onset osteoporosis. We reported a 33-year-old male patient presenting a history of recurrent nephrolithiasis and early onset osteopenia in the lumbar spine and femur. He was tested, through next generation sequencing (NGS), by using a customized multigenic panel containing 33 genes, whose mutations are known to be responsible for the development of congenital parathyroid diseases. Two further genes, *SLC34A1* and *SLC34A3*, encoding two sodium–phosphate cotransporters, were additionally tested. A novel germline heterozygous mutation was identified in the *SLC34A1* gene, c.1627G>T (p.Gly543Cys), currently not reported in databases of human gene mutations and scientific literature. *SLC34A1* germline heterozygous mutations have been associated with the autosomal dominant hypophosphatemic nephrolithiasis/osteoporosis type 1 (NPHLOP1). Consistently, alongside the clinical features of NPHLOP1, our patient experienced recurrent nephrolithiasis and lumbar and femoral osteopenia at a young age. Genetic screening for the p.Gly453Cys variant and the clinical characterization of his first-degree relatives associated the presence of the variant in one younger brother, presenting renal colic and microlithiasis, suggesting p.Gly453Cys is possibly associated with renal altered function in the NPHLOP1 phenotype.

## 1. Introduction

Phosphorus is one of the most represented elements in the human body, mainly in the form of phosphate salts. Phosphates are involved in numerous intracellular processes, such as signal transduction, energy production, pH maintenance, phospholipid structure, and nucleic acid synthesis, but about 85% of phosphate is located in bone tissue, where it represents one of the major components of the mineralized extracellular matrix [1]. Extracellular and circulating phosphate levels are hormonally regulated, mainly through the regulation of phosphate renal reabsorption, mediated by a family of sodium–phosphate cotransporters expressed on the cell membrane of the proximal tubules of the kidney, which allow for the reabsorption of 70–80% of filtered phosphate [2]. Sodium–phosphate cotransporters are classified in three types, Na-Pi-I, Na-Pi-II, and Na-Pi-III, respectively encoded by the *SLC17*, *SLC34,* and *SLC20* family genes [3]. Sodium-dependent phosphate transport protein 2a (NaPi-IIa), encoded by the solute carrier family 34 member 1 (*SLC34A1*) gene, and sodium-dependent phosphate transport protein 2c (NaPi-IIc), encoded by the solute carrier family 34 member 3 (*SLC34A3)* gene, are specifically expressed in the kidneys [4].

Germline heterozygous mutations of the *SLC34A1* gene have been associated with a rare Mendelian clinical phenotype, the nephrolithiasis/hypophosphatemic osteoporosis type 1 (NPHLOP1), which is variably characterized by hyperphosphaturia, hypophosphatemia, recurrent kidney stones, skeletal demineralization, bone pain, and early onset osteoporosis [5]. Germline homozygous or compound heterozygous mutations of the *SLC34A3* gene have been associated with an autosomal recessive rare disease, namely hypophosphatemic rickets with hypercalciuria, which is characterized by hypophosphatemia secondary to renal phosphate wasting, radiographic and/or histologic evidence of rickets, limb deformities, muscle weakness, and bone pain [6,7].

Here, we report the case of a 33-year-old male patient, presenting with a history of recurrent nephrolithiasis and early onset osteopenia in the lumbar spine and femur firstly assessed at the age of 31 years, in whom we identified a novel germline heterozygous mutation of the *SLC34A1* gene.

## 2. Case Presentation

### 2.1. Clinical Case

Informed written consents were obtained from the proband and his first-degree relatives for the publication of this case report.

The proband was a male patient, first referred to our attention in September 2019 at the age of 33, reporting a clinical history of relapsing renal colic, starting at the age of 22 years, in non-bilateral multiple nephrolithiasis, which was treated by surgery in 2014 and 2017. Surgery resulted to be non-resolutive, with the persistence of lithiasis. The presence of a right renal cyst of 15 × 12 × 13 cm was reported. Hypertension was also assessed.

A dual-energy X-ray absorptiometry (DXA) assessment, performed on January 2017 (at the age of 31 years), showed a reduced bone mineral density (BMD) for his age, both at the lumbar spine and the left femoral neck (the Z-score was −2.1 and −2.2, respectively).

At the time of his first visit to our ambulatory in September 2019, the patient was in treatment with potassium citrate and magnesium citrate (one sachet/day), calcifediol (30 drops/week), 5 mg of amlodipine (one tablet/day), and 2.5 mg of bisoprolol fumarate (one tablet/day).

Biochemical screening showed that serum phosphate was constantly at the lower limit of the reference range, and also showed slightly elevated values of total calcium and parathyroid hormone (PTH). A deficiency of 25-(OH)-vitamin D was initially reported, showing a normalization during prolonged supplementation with calcifediol in the four years of follow-up. At the time of the first visit at our ambulatory, the proband showed increased urinary excretion of both calcium (317 mg/24 h; reference range: 100–300 mg/24 h) and phosphorus (1550 mg/24 h; reference range: 400–1300 mg/24 h).

After the assessment of hypercalciuria in September 2019, the patient was administered with 25 mg of chlorthalidone (one cp/day) and 200 mg of clodronate (one intramuscular injection every 2 weeks), with the interruption of 30 drops/week of calcifediol.

At the next clinical check-up in January 2021, at the age of 35 years, the patient was reported to have a recurrent bilateral nephrolithiasis, and an increased size and number of kidney stones, with respect to the previously performed abdominal ultrasound examination. He showed normalization of calciuria and normal phosphaturia, with concomitant and slightly increased values of serum PTH and total calcium, reduced values of 25-(OH)-vitamin D and serum phosphate, and increased values of urinary free cortisol.

A DXA evaluation confirmed an important reduction in BMD for the patient’s age, both at the lumbar spine and the left femoral neck (the Z-scores totaled −2.4 and −1.7, respectively).

Given the increased values of PTH in the proband and his mother, and following the suspicion of a congenital familial primary hyperparathyroidism, the patient was screened for multiple endocrine neoplasia type 1 (MEN1)-associated main tumors.

In March 2021, a glucose load curve was performed to exclude the presence of an insulinoma:

− time 0′ glucose 99 mg/dL (reference value < 110) and insulin 19.5 IU/mL, range 2.6–24.9);
− time 60′ glucose 172 mg/dL (reference value < 110) and insulin 19.6 IU/mL, range 2.6–24.9);
− time 120′ glucose 152 mg/dL (reference value < 110) and insulin 21.1 IU/mL, range 2.6–24.9).

In July 2021, a CT scan of the upper and lower abdomen was performed, evidencing multiple stones in both kidneys, and an increase in the dimensions of some stones, compared to the previous screening. No stones were detected in the right ureter or in the bladder. The CT scan showed the presence of a left pyeloureteral stent and confirmed the presence of a voluminous right renal cyst, measuring 15 × 12 × 13 cm. No lesions were found in the pancreas, the duodenum, or the liver.

The therapy was adjusted to potassium citrate, magnesium citrate, 25 mg of chlorthalidone (half cp/day), calcifediol (15 drops/week), 25 mg of neridronate (one intramuscular injection/month), monobasic phosphate and dibasic potassium phosphate (one sachet/2 times a day), 5 mg of amlodipine (one cp/day), and 2.5 mg of bisoprolol fumarate (one cp/day).

In January 2022, brain and pituitary MRIs were performed, both with and without a contrast medium, to exclude the presence of pituitary adenoma, a common manifestation of MEN1 syndrome. The MRI analyses showed a normal adenohypophysis and a pituitary stalk of normal caliber, which was not deviated. The neurohypophysis did not show the physiological T1 hyperintensity.

In 2022, the patient underwent surgery for the laparoscopic removal of the right renal cyst. The same year, high-resolution peripheral quantitative computed tomography (HR-pQCT) of the non-dominant distal radius showed a clearly reduced bone volume density, compared to the mean reference value, while the trabecular thickness and cortical porosity were both within the range of the mean reference value.

The patient is currently undergoing tests for an evaluation of hypercortisolism of a probable secondary nature.

### 2.2. Family Screening

**Figure 1:**
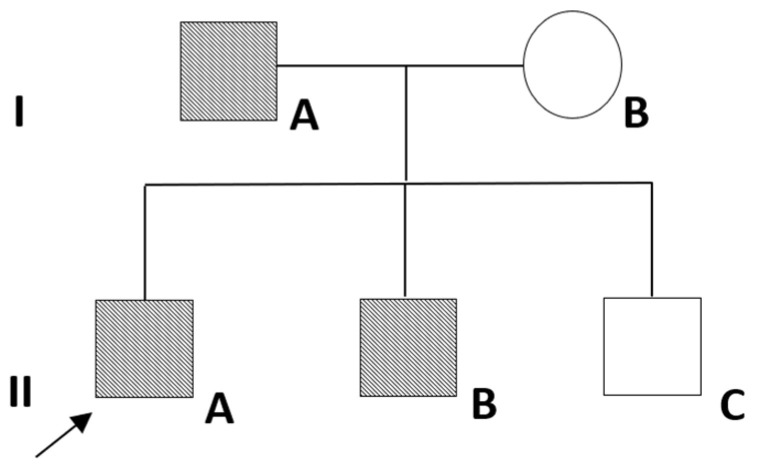
Family pedigree. Roman numerals on the left identify the two different generations. Round symbols are indicative of females and squared symbols represent males. The black arrow indicates the index case (IIA). IA is the father, IB is the mother, and IIB and IIC are two younger brothers of the proband. Black-striped symbols indicate *SLC34A1* mutation carriers, while white symbols indicate individuals not bearing the *SLC34A1* mutation.

The family pedigree of the proband is represented in Figure 1.

A study of the family history reported that one of the two younger brothers of the proband (IIB) had recurrent (three) renal colic by the age of 30 years, as well as diffuse muscular and tendon pain.

The available data on the biochemical measurements of phosphorus and calcium metabolism in the serum and urine of the first-degree relatives (the mother, father, and two younger brothers) were recovered.

The father (IA) and the youngest brother (IIC) showed normal levels of serum calcium, serum phosphate, and PTH, as well as normal calciuria and phosphaturia.

The mother (IB) showed significantly increased PTH values, associated with normocalcemia and an insufficiency of 25-(OH)-vitamin D. Her urinary excretions of calcium and phosphate were normal, as well as the serum levels of both of these ions.

The other brother (IIB) presented with hypercalciuria, associated with normal values of PTH, serum calcium, serum phosphate, and phosphaturia, and an insufficiency of 25-OH-vitamin D, in the presence of a history of frequent renal colic.

The mother and the two brothers underwent ultrasound analyses of their abdomens, revealing no signs of lithiasis in the mother and brother IIC, while brother IIB was found to have bilateral microlithiasis and a kidney stone measuring 5.5 mm in the right kidney, at the age of 35 years. No abdominal screening was available for the father.

Brother IIC had a total body DXA analysis performed at the age of 30 years, showing a normal value of BMD for his age (the Z-score was −0.6). No DXA screenings were available for the mother, the father, or brother IIB.

The genetic, biochemical, and clinical characteristics of the proband and his first-degree relatives are summarized in Table 1.

### 2.3. Genetic Testing

The elevated PTH in the proband, associated with slight hypercalcemia, and the recurrent elevated PTH in the mother were suspected to be signs of a possible familial congenital primary hyperparathyroidism, and he was initially tested in February 2022 by next generation sequencing (NGS), using a customized multigenic panel (named “parathyroid congenital diseases panel”), containing 33 genes whose mutations are known to be responsible for the development of congenital parathyroid diseases, which was designed and developed by the scientific and technical collaboration between the Fondazione FIRMO Onlus and the Personal Genomics SRL. Two additional genes, *SLC34A1* and *SLC34A3*, encoding two sodium–phosphate cotransporters, were tested, by NGS screening, in May 2022.

A venous blood sample was collected from the proband for the genetic analysis. The genomic DNA was extracted by Qiaamp mini kit (Qiagen GmbH, Hilden, Germany). The generation of DNA libraries and target enrichment were performed according to the Kapa HyperCap protocol (Roche, Basilea, Switzerland). The DNA target regions included all the coding regions and 20-base-length intron–exon junctions. The target-enriched libraries were sequenced using a MiSeq Reagent Kit v2 on the MiSeq platform (Illumina, San Diego, CA, USA) with a “paired-end” protocol with reads measuring 150 bp in length. Raw reads were filtered using fastp (version 0.20.1) to remove adapters, low-quality reads, and low-quality bases from the reads. The filtered reads were then mapped on the HG38 human reference genome using BWA-mem (version 0.7.17-r1188). Polymerase Chain Reaction (PCR) duplicate fragments were removed by MarkDuplicates, and base quality recalibration scores were computed by BaseRecalibrator (GATK version 4.1.1.0.). Single nucleotide variants and small intra–exonic insertions/deletions were called using HaplotypeCaller and hard-filtered with VariantFiltration (GATK version 4.1.1.0.). Large intragenic duplications or deletions were assessed in the genes of interest, using a set of human genomic regions that are stable for the number of copies as references.

Identified rare variants were classified and selected according to the standards and guidelines for the interpretation of human sequence variants of the American College of Medical Genetics and Genomics (ACMG), using Expert Variant Interpreter—eVAI software (eVAI version 2.5; enGenome, Pavia, Italy).

The NGS screening did not find the presence of any pathogenic or potentially pathogenic variant in the 33 analyzed genes associated with congenital parathyroid diseases or in the *SLC34A3* gene.

**Figure 2:**
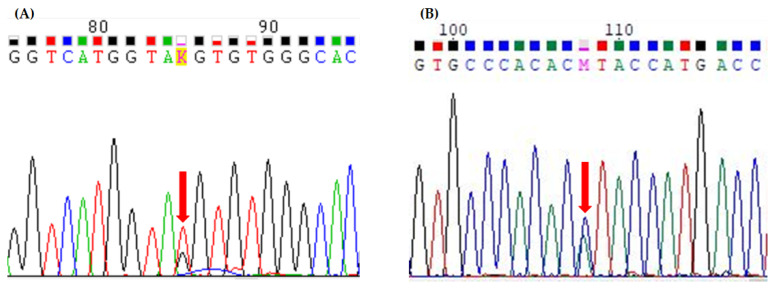
Forward panel (**A**) and reverse panel (**B**) sequences of exon 13 of the *SLC34A1* gene in our case report, obtained by the PCR-based Sanger’s sequencing technique, showing the presence of the c.1627G>T heterozygous substitution (p.Gly543Cys). Red arrows indicate the mutated nucleotide.

A germline heterozygous G>T transversion at nucleotide 1627 in the *SLC34A1* gene (NM_003052.5), leading to a glycine–cysteine substitution at amino acid 543 (c.1627G>T; p.Gly543Cys) (NP_003043.3), was identified by the NGS analysis of the *SLC34A1* gene, and confirmed by PCR-based Sanger’s sequencing of the exon 13 (Figure 2).

After the identification of the *SLC34A1* mutation, the genetic screening for this specific variant was extended to the first-degree relatives of the proband (his mother, father and the two brothers), through a PCR-based direct sequencing of the *SLC34A1* exon 13 by Sanger’s method. The p.Gly543Cys variant was identified in the father (IA) and in one of the two brothers (IIB) (Figure 1).

Currently, the p.Gly543Cys mutation is not reported in databases of human gene mutations [GnomAD, ClinVar, or Human Gene Mutation Database (HGMD)], in the National Center for Biotechnology Information-Single Nucleotide Polymorphism (NCBI SNP) database, or published in the scientific literature, and the data about its frequency in humans are not available; thus, we classified it as a novel rare variant. According to the American College of Medical Genetics (ACMG) criteria for the classification of human gene variants, the p.Gly543Cys resulted as PM2 (absent from controls in Exome Sequencing Project, 1000 Genomes, or ExAC), and PP3 (multiple computational programs predictive of the impact of a gene mutation on the structure and/or function of the encoded protein supported a possible deleterious effect of the c.1627G>T point variant on NaPi-IIa protein), and was, thus, classified as a rare variant of uncertain clinical significance (VUS) [8].

The results from an in silico predictive assessment of the c.1627G>T mutation’s impact on NaPi-IIa structure and function are summarized in Table 2.

The human amino acid Gly543 is conserved in rat, mouse, rabbit, and sheep (Table 3).

## 3. Discussion

The NGS-based genetic screening of a set of genes, involved in the functionality of parathyroid glands and in the regulation of calcium and phosphate homeostasis, identified a novel germline heterozygous missense mutation of the *SLC34A1* gene (p.Gly543Cys) in a young male patient with recurrent nephrolithiasis and early onset osteopenia.

**Figure 3:**
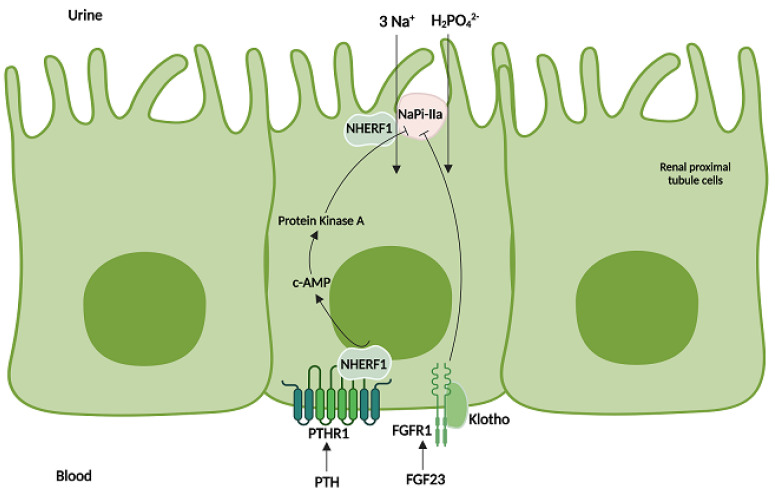
Function and regulation of NaPi-IIa. The NaPi-IIa cotransporter is located in the apical brush border of the renal proximal tubule epithelial cells, where it mediates the reabsorption of phosphate and sodium from the urine to the blood. Reabsorption of phosphate from the renal tubule is blocked by parathyroid hormone (PTH) and fibroblast growth factor 23 (FGF23), which act upon their respective receptors, PTHR1 and FGFR1, to down-regulate the number of NaPi-IIa luminal transmembrane channels. The figure was created using BioRender.com.

The *SLC34A1* gene encodes the renal-specific sodium–phosphate cotransporter NaPi-IIa, a 639 amino acid protein located at the apical brush border of the proximal tubule of the kidneys, which is the most important regulator of phosphate renal reabsorption (Figure 3).

Knocked-out mice for the *Slc34a1* gene showed renal phosphate wasting, hypophosphatemia, hypercalciuria, and skeletal abnormalities, confirming the importance of the NaPi-IIa cotransporter in phosphate homeostasis [9].

The NaPi-IIa cotransporter is composed of seven helical transmembrane domains, located at amino acids 100–121, 141–160, 348–370, 403–431, 469–491, 511–534, and 540–561. The mutation identified in our patient is located within the seventh transmembrane domain of the channel.

The functional characterization of naturally occurring mutations in the human *SLC34A1* gene, expressed in Xenopus laevis oocytes, showed the reduced phosphate affinity of the mutated NaPi-IIa protein (p.Ala48Phe, p.Val147Met) [4], a reduction in the sodium-induced current of inorganic phosphate through the mutated NaPi-IIa cotransporter (p.Ala48Phe, p.Val147Met, c.91del7, p.Ile456Asn, p.Arg512Cys) [2,5,10], and disturbed trafficking to the plasma membrane of the mutated NaPi-IIa protein, with a loss of phosphate transport activity (p.Ile456Asn, p.Arg512Cys, c.91del7) [2,11]. None of the functionally analyzed *SLC234A1* mutations were located within the same protein domain of the p.Gly543Cys.

Heterozygous mutations of the *SLC34A1* gene have been associated with the NPHLOP1, a rare autosomal dominant clinical condition characterized by idiopathic hyperphosphaturia and subsequent hypophosphatemia that contributes to the development of nephrolithiasis and early bone demineralization. Prié et al. [5] described a male patient with recurrent renal stones and a woman with evidence of idiopathic bone demineralization bearing two distinct germline heterozygous missense mutations of the *SLC34A1* gene, the p.Ala48Phe and the p.Val147Met, respectively. More recently, the p.Ile456Asn mutation was found in a 26-year-old male patient with hypophosphatemia, bilateral kidney stones, and an autosomal dominant family history of nephrolithiasis [2], the p.Asn227Ser mutation was identified as segregating with disease occurrence in a pedigree with persistent hypophosphatemia, progressive musculoskeletal pain, and weakness in the lower extremities [12], and the p.Ser585Pro mutation was found in a 38-year-old woman with hypophosphatemic kidney stones with osteoporosis, and pain and weakness of the lower limbs [13]. The clinical phenotype of our proband, presenting with recurrent nephrolithiasis and severe osteopenia at a young age, is compatible with NPHLOP1 and with the clinical phenotypes previously described in patients with *SLC34A1* heterozygous mutations [5,13], suggesting that the identified p.Gly543Cys variant could be responsible for the development of clinical manifestations at the kidneys and the skeleton. Moreover, the identification of the same mutation in one younger brother with a history of recurrent renal colic in presence of bilateral microlithiasis may further indicate a direct role of this novel identified variant in causing nephrolithiasis.

## 4. Conclusions

In our familial case, the genetic screening of the second brother of the proband was observed to bear the same mutation that was carried by the propositus. The brother was experiencing renal colic and nephrolithiasis, which suggested that p.Gly453Cys could be a putative marker for NPHLOP1 mediated by renal functions. Patients with similar symptoms and clinical manifestations should undergo a genetic diagnosis, as performed by the current study, for mutation detection of the *SLC34A1* gene and other genes encoding sodium-dependent phosphate transport proteins.

## Figures and Tables

**Table 1:** Genetic, biochemical, and clinical characteristics of the proband and his first-degree relatives.

Patient Identificative Code	Genetic Testing	Serum Calcium	Serum Phosphate	PTH	25-(OH)-Vitamin D	24h Urinary Excretions of Calcium	24 h Urinary Excretions of Phosphate	Ultrasound Analysis of the Abdomen	Clinical History of Kidneys	DXA Scan
IIA (proband)	Positive for the p.Gly543Cys (*SLC34A1*)	Slightly elevated	Normal value	Slightly elevated	Deficiency assessed at 31 years old and insufficiency at 33 and 35 years old (normalized after 4 years of supplementation with calcifediol at 36 years old)	Slight hypercalciuria (normalized after therapy)	Hyperphosphaturia (normalized after therapy)	At 33 years old: Clear evidence of bilateral nephrolithiasis At 35 years old: Bilateral nephrolithiasis, and an increase in both the size and number of kidney stones	History of relapsing renal colic by the age of 22. Non-bilateral multiple nephrolithiasis, treated twice by surgery at the ages of 28 and 31 years. Persistence of nephrolithiasis after two surgeries.	At 31 years old: Z-score: −2.1 (L1-L4) −2.2 (femur neck) At 35 years old: Z-score: −2.4 (L1-L4) −1.7 (femur neck)
IA (father)	Positive for the p.Gly543Cys (*SLC34A1*)	Normal value	Normal value	Normal value	Not reported	Normal value	Normal value	Not performed	No history of either altered kidney function or nephrolithiasis	Not performed
IB (mother)	Negative for the p.Gly543Cys (*SLC34A1*)	Normal value	Normal value	Significantly increased	Insufficiency	Normal value	Normal value	At 58 years old: No sign of nephrolithiasis	No history of either altered kidney function or nephrolithiasis	Not performed
IIB (brother)	Positive for the p.Gly543Cys (*SLC34A1*)	Normal value	Normal value	Normal value	Insufficiency	Slight hypercalciuria	Normal value	At 35 years old: Bilateral microlithiasis and a kidney stone measuring 5.5 mm in the right kidney	Recurrent (three) renal colic by the age of 30 years	Not performed
IIC (brother)	Negative for the p.Gly543Cys (*SLC34A1*)	Normal value	Normal value	Normal value	Not reported	Normal value	Normal value	At 28 years old: No sign of nephrolithiasis	No history of either altered kidney function or nephrolithiasis	At 31 years old: Z-score: −0.6 (total body)

**Table 2:** In silico predictive assessment of c.1627G>T mutation’s impact on NaPi-IIa structure and function.

Nucleotide Change (Ref. Sequence NM_003052.5)	Amino Acid Change (Ref. Sequence NP_003043.3)	Amino Acid Change Score (Grantham Matrix)	Mutation Taster 2	PolyPhen-2	SIFT
c.1627G>T (g.18759G>T)	p.Gly543Cys (G543C)	159	Disease-causing	Probably damaging	0.01

Footnotes: The Grantham matrix score is a prediction of the effect of substitutions between two amino acids based on chemical properties, including polarity and molecular volume, which are characterized into classes of increasing chemical dissimilarity: conservative (0–50), moderately conservative (51–100), moderately radical (101–150), or radical (≥151). Polymorphism Phenotyping v2 (PolyPhen-2) is a tool which predicts the possible impact of an amino acid substitution on the structure and function of a human protein, using straightforward physical and comparative considerations and structural features, such as amino acid atomic contacts and solvent accessibility. Empirically determined cut-offs are used to predict if the substitution is “probably damaging”, “possibly damaging” or “benign”. Sorting Intolerant From Tolerant (SIFT) is an algorithm which predicts whether an amino acid substitution will affect protein function, based on sequence homology and the physical properties of amino acids. A SIFT score of less than 0.05 is predicted to be deleterious, while a SIFT score greater than or equal to 0.05 is predicted to be tolerated.

**Table 3:** Evolutionary conservation of the human Gly543 residue.

Species	GeneBank Protein ID	Amino Acids	Amino Acid Sequence																				
*Homo sapiens* (Human)	AAA36354.1	639	I	S	M	A	G	W	Q	V	M	V	**G**	V	G	T	P	F	G	A	L	L	A
*Rattus norvegicus* (Rat)	AAC37608.1	637	I	S	M	A	G	W	Q	A	M	V	**G**	V	G	T	P	F	G	A	L	L	A
*Mus musculus* (Mouse)	AAC42026.1	637	I	S	M	A	G	W	Q	A	M	V	**G**	V	G	T	P	F	G	A	L	L	A
*Oryctolagus cuniculus* (Rabbit)	AAA77682.1	642	I	S	M	A	G	W	R	A	M	V	**G**	V	G	A	P	F	G	A	L	L	A
*Ovis aries* (Sheep)	CAA04715.1	639	L	S	M	A	G	W	R	A	M	G	**G**	V	G	A	P	F	G	A	L	L	A

Footnote: The conserved Glycine (G) residue is highlighted in bold.

## Data Availability

Data contained within the article are available from the corresponding author on reasonable request.
